# ADDENDUM: Peroxisome proliferator-activated receptor γ inhibits follicular and anaplastic thyroid carcinoma cells growth by upregulating p21^*Cip1/WAF1*^ gene in a Sp1-dependent manner

**DOI:** 10.1530/ERC-07-0272a

**Published:** 2026-09-07

**Authors:** D Bonofiglio, H Qi, S Gabriele, S Catalano, S Aquila, M Belmonte, S Ando

**Affiliations:** ^1^ Department of Pharmaco-Biology, University of Calabria, Arcavacata di Rende (Cosenza), Italy; ^2^ Centro Sanitario, University of Calabria, Arcavacata di Rende (Cosenza), Italy; ^3^ Department of Cellular Biology, University of Calabria, Arcavacata di Rende (Cosenza), Italy; ^4^ Faculty of Pharmacy, University of Calabria, Arcavacata di Rende (Cosenza), Italy

With the agreement of the Editor-in-Chief, the Deputy Editor, and the publisher Bioscientifica Ltd, *Endocrine-Related Cancer* is publishing an Addendum regarding an article that appeared in volume 15, part 2, pages 545–557 (https://doi.org/10.1677/ERC-07-0272).

In March 2026, the journal was alerted to potential image manipulation in Fig. 6, given on page 553. The authors state that the data presented in the figures were authentic and represent independent experimental results.

Due to several factors, some original raw data (including uncropped digital image files) are no longer available. However, the authors have provided the original western blot films corresponding to the experiment shown in Fig. 6B, including the immunoprecipitation data involving PPARγ and Sp1. The original western blots corresponding to Fig. 6B are given in full below:

**Figure:**
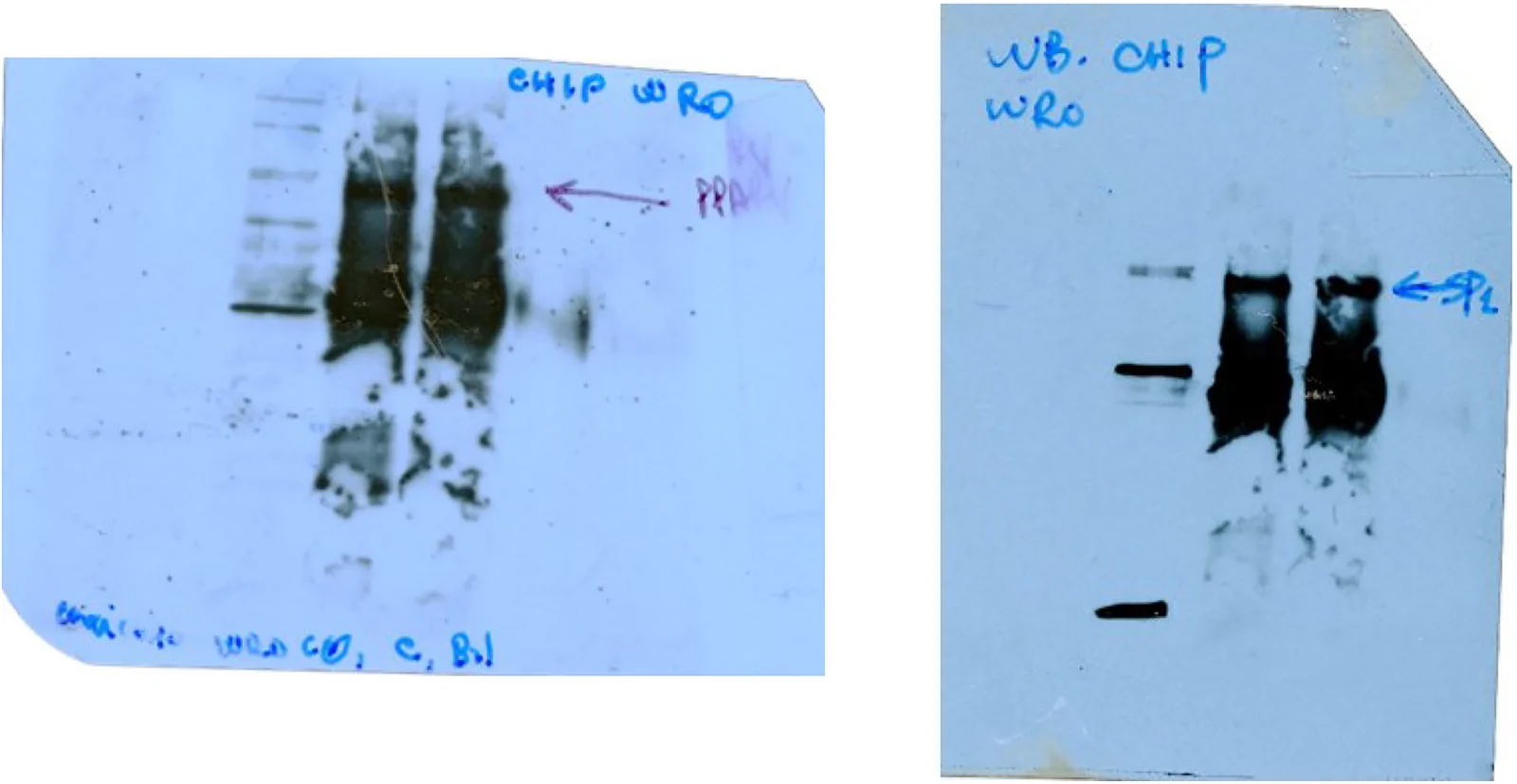


This clarification does not impact the results, interpretation, or conclusions of the original paper.

The original Fig. 6 is given in full below:

**Figure 6:**
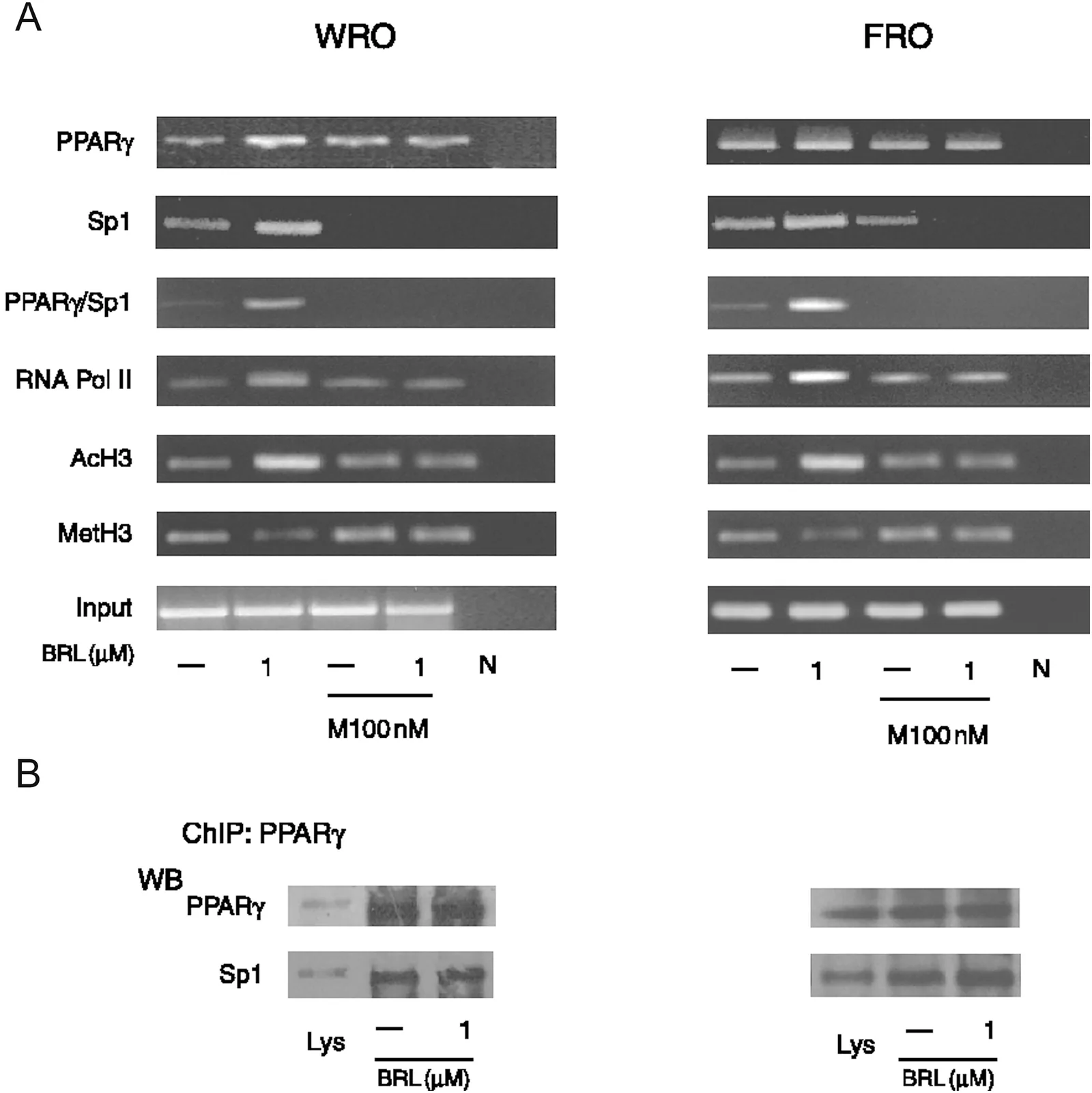
Functional interaction between PPARγ and p21^*Cip1/WAF1*^ promoter in ChIP and ReChIP assays. (A) WRO and FRO cells were treated as indicated, then cross-linked with formaldehyde, and lysed. The soluble chromatin was immunoprecipitated with the anti-PPARγ, anti-Sp1, anti-RNA Pol II, anti-acetyl histone H3 (AcH3), and anti-trimethyl histone H3 (MetH3) antibodies. Chromatin immunoprecipitated with the anti-PPARγ antibody was re-immunoprecipitated with the anti-Sp1 antibody. The p21^*Cip1/WAF1*^ promoter sequence containing Sp1 was detected by PCR with specific primers, as described in Materials and methods. To control input DNA, the p21^*Cip1/WAF1*^ promoter was amplified from 30 μL initial preparations of soluble chromatin (before immunoprecipitations). N, negative control provided by PCR amplification without DNA sample; M, mithramycin. (B) Immunoblots of PPARγ and Sp1 from WRO and FRO cells treated with BRL 1 μM in which chromatin was immunoprecipitated with the anti-PPARγ antibody. Lys, cell lysates.

